# Gastroesophageal reflux disease in patients with long standing type 1 diabetes mellitus: utility of two self-report questionnaires in a multifactorial disease

**DOI:** 10.25100/cm.v48i3.2801

**Published:** 2017-09-30

**Authors:** Emmanuel Marin Valdez-Solis, Claudia Ramírez-Rentería, Aldo Ferreira-Hermosillo, Mario Molina-Ayala, Victoria Mendoza-Zubieta, Víctor Rodríguez-Pérez

**Affiliations:** 1 Servicio de Endocrinología. Hospital de Especialidades, Centro Médico Nacional Siglo XXI , Instituto Mexicano del Seguro Social. Ciudad de México, México.; 2 Unidad de Investigación Médica en Endocrinología Experimental. Hospital de Especialidades, Centro Médico Nacional Siglo XXI, Instituto Mexicano del Seguro Social. Ciudad de México, México.; 3 Facultad de Psicología. Universidad Nacional Autónoma de México. Ciudad de México, México.

**Keywords:** gastroesophageal reflux, type 1 diabetes mellitus, surveys and questionnaires, Reflujo gastroesofágico, diabetes mellitus tipo 1, encuestas y cuestionarios

## Abstract

**Background:**

Gastroesophageal pathologies are common and multifactorial in patients with type 1 diabetes (T1DM). The evaluation with endoscopy and 24 h pH esophageal monitoring is expensive and not always available in all medical centers, especially in developing countries so more cost-effective algorithms for diagnosis are required. Clinical questionnaires are easy to apply but its utility for gastroesophageal reflux disease screening in patients with long standing T1DM must be analyzed.

**Objective:**

To evaluate the utility of the FSSG and Carlsson-Dent (CDQ) questionnaires to detect the frequency of gastroesophageal reflux disease in patients with T1DM.

**Methods:**

Analytic cross-sectional study, included 54 randomly selected patients from the T1DM clinic in our hospital. Before their routine evaluation, were asked to answer FSSG and CDQ questionnaires, classifying them as positive with a score >8 or >4, respectively. we associated and compared the clinical and biochemical characteristics between patients with or without gastroesophageal reflux detected through questionnaires.

**Results:**

Median age was 29 years (22-35), 67% were female (median of 16 years from diagnosis). In 39% of the patients FSSG was positive, CDQ was positive in 28%. A total of 71% of patients were taking medications to treat non-specific gastric symptoms. The concordance between questionnaires was 65% (*p*: <0.001). Those patients with tobacco consumption as well as those with poor glycemic control were more likely to score positive in either questionnaire.

**Conclusions:**

Patients T1DM had a high prevalence of gastroesophageal reflux disease. In those patients FSSG questionnaire detected a higher number of patients in comparison with CDQ.

## Resumen

**Introducción:**

Las patologías gastroesofágicas son comunes y multifactoriales en pacientes con diabetes tipo 1 (DM1). La evaluación por medio de panendoscopia y pHmetría es costosa y difícil de realizar en todos los centros de atención, por lo que se requieren algoritmos rentables para su diagnóstico. Existen cuestionarios sencillos y autoaplicables que pueden ser útiles para el diagnóstico de enfermedad por reflujo gastroesofágico en los pacientes con DM1.

**Objetivo:**

Evaluar la utilidad de los cuestionarios FSSG y Carlsson-Dent (CDQ) para detectar la enfermedad por reflujo gastroesofágico (ERGE) en pacientes con DM1.

**Métodos:**

Estudio transversal, se incluyeron 54 pacientes, elegidos al azar de la clínica de DMT1. Previo a la consulta, se les solicitó contestaran los cuestionarios FSSG y el CDQ, considerándose positivos para diagnóstico de ERGE los puntajes >8 y >4, respectivamente. Se analizaron y compararon las características bioquímicas y clínicas entre los pacientes con y sin síntomas de ERGE detectada por medio de los cuestionarios.

**Resultados:**

Los pacientes estudiados tenían edad de 29 años (22-35), 67% fueron mujeres, (mediana de diagnóstico de 16 años). El 39% de los pacientes tenían ERGE detectado mediante FSSG y 28% utilizando el cuestionario CDQ. El 71% de los pacientes reportó uso de medicamentos para reflujo. La concordancia entre ambos cuestionarios fue del 65% (*p*: <0.001). Pacientes que consumen tabaco y con descontrol glucémico, tenían más probabilidades de positividad en cualquier cuestionario.

**Conclusiones:**

Existe una alta prevalencia de ERGE en los pacientes con DM1. En esta población el cuestionario FSSG detectó a un mayor número de pacientes en comparación con el CDQ.

## Introduction

Gastroesophageal Reflux Disease (GERD) occurs when the stomach contents leak backwards from the stomach into the esophagus, causing symptoms that impair the quality of life or in extreme cases may be the cause of other complications. Its prevalence varies from 10 to 40% in the adult population, but may be influenced by the population's characteristics, type of diet and the presence of other comorbidities ^1^. Gastrointestinal diseases are common in patients with diabetes mellitus, as well as the symptoms of dysphagia, heartburn and regurgitation ^2^. Detecting GERD in patients with diabetes is mainstay since it decreases oral tolerance to multiple medications, including hypoglycemic agents which may render their glucose control difficult and usually impairs the quality of life ^3^. Furthermore due to its non-specific symptoms, it could go unnoticed for many years.

Proper identification of GERD symptoms ^4^ in patients with type 1 diabetes (T1D) is complicated because long-standing poorly controlled diabetes and its comorbidities may present with similar symptoms ^5^. Autonomic neuropathy is one of the most common diagnosis in these patients and it is of great concern in patients that have been treated for diabetes for more than 10 years, since it may be present despite an adequate glycemic control. Autonomic neuropathy is highly prevalent and irreversible and the symptoms can be easily mistaken for GERD or viceversa.

Patients with T1D may develop other diseases such as autoimmune gastritis, celiac or Crohn's disease whose symptoms could be confused with GERD. In order to properly diagnose those diseases, it's necessary to perform endoscopies, serum identification of autoantibodies and follow specific diagnostic algorithms by specialized gastroenterologists. These studies can be annoying to the patient, costly and most health systems limit them to severe cases or give priority to patients with other positive diagnostic tests. Therefore, it would be more practical to first rule out the diagnosis of GERD by means of an effective and easy-to-use clinical tool.

In our country, access to the diagnostic tools and specialized medicine is very restricted due to the large volume of patients and the few available specialists, therefore gastroenterologists suggest the use of clinical scales as indicators of GERD. The clinical scales have been developed to allow proper identification of the disease. Carlsson et al., developed a questionnaire (CDQ) for this purpose and validated their findings with endoscopy and 24 h pHmetry ^6^. This questionnaire is self-completed and scores greater than 4 points have a sensitivity of 92% and a specificity of 19% for GERD screening. Later, Kusano *et al*., developed the FSSG questionnaire (Frequency Scale for the Symptoms of GERD), arguing that CDQ detects symptoms of GERD but not exclusive of it, and that it is not useful for assessing the severity of the GERD or the treatment's response. According these authors, a FSSG score greater than 8 points has a sensitivity of 62% and specificity of 59% for the diagnosis of GERD, as corroborated by endoscopy ^7^.

Both questionnaires have been widely used and validated in different populations, including patients from our country ^8^ ^-^ ^10^ and patients with type 2 diabetes mellitus ^11^ ^,^ ^12^. However, they have not been used in patients with T1D. The objective of this study was to evaluate the prevalence and severity of GERD in patients with T1D using a simple and self-fulfilling tool.

## Materials and Methods

We performed a cross-sectional analytical study in a group of patients randomly selected from the T1D Clinic of the Hospital de Especialidades del Centro Médico Nacional Siglo XXI of the Instituto Mexicano del Seguro Social (IMSS) from February to May 2015. All patients have met the T1D diagnosis criteria of the American Diabetes Association (ADA) ^13^ defined as the detection of anti-GAD65 or anti-IA2 autoantibodies and a serum fasting C-peptide below of the inferior limit for our population ^13^. This study not restricted their selection by age or weight.

The inclusion criteria were a time since diagnosis of at least five years, visual ability to read the questionnaire questions and at academic capacity to understand the questionnaire without help. Patients with a history of gastric surgery, previous diagnosis with endoscopy, unintentional weight loss, severe or progressive dysphagia, or gastrointestinal bleeding were excluded. Clinical and biochemical data were recorded at the time of initial assessment. All patients completed the FSSG ^10^ (Annex 1) and CDQ ^14^ (Annex 2) questionnaires validated in Spanish. A CDQ questionnaire with a score greater than 4 or FSSG with a score greater than 8, were considered positive for the detection of GERD. All questionnaires were applied by the principal investigator (EVS) and analyzed by a blinded investigator (CRR) with a κ= 0.7 (concordance), *p*: <0.001.

The Local Research and Ethics Committee on Health Research approved the protocol. The objectives of the study were fully explained to the participants, who gave their written informed consent. The procedures followed ethical standards according to the Declaration of Helsinki of 1975, revised in 2013.

### Biochemical evaluation

Laboratory studies were requested after an 8-hour fasting period. Glucose, cholesterol, HDL cholesterol (HDL-c) and triglycerides were determined using spectrophotometry technique ^15^, using commercial kits. To obtain serum HDL-c concentration, an enzymatic precipitation test with polyethylene glycol and dextran sulfate ^16^ was performed and then analyzed with the same photocolorimetric technique as for cholesterol. Glycated hemoglobin (HbA1c) was analyzed by immunoassay. The concentration of LDL cholesterol (LDL-c) was calculated using the Friedewald formula ^17^.

### Statistical analysis

Data were analyzed using the statistical package STATA(r) version 11. A Kolmogorov-Smirnov test was used to assess normality. Results are expressed as medians with interquartile ranges (RI). The results were evaluated by stratified analysis, searching for associations between the quantitative variables with a Mann-Whitney U test or Student's t test, and for qualitative variables using χ^2^ or Fisher's test. Receiver Operating Characteristics (ROC) curves of the different scales were performed to obtain the best cutoff point (with 95% confidence intervals) for the detection of GERD. A *p* <0.05 was considered as statistically significant.

## Results

### Baseline characteristics

Fifty-four patients met the inclusion criteria and completed the questionnaire, 36 were women (67%). The median age of the studied group was 29 years (23-35 years), while the mean evolution time was 16 years (range 10-22 years). All patients had more than 5 years of diagnosis of type 1 diabetes and 72% more than 10 years of diagnosis (Table 1). Their associated comorbidities were: another autoimmune disease in 43% (mainly hypothyroidism); 20.4% had dyslipidemia (previously detected with hypertriglyceridemia or decreased HDL-c levels according to sex, or were in treatment); 18.4% had previous diagnosis of hypertension and 14% had chronic kidney disease (validated by the KDOQI scale using the glomerular filtration rate).

**Table 1:** Baseline characteristics of 54 patients with T1D with and without GERD.

Variable	Total population (n= 54)	Without GERD symptoms (n= 32)	Positive* (n= 22)	p**
Age, years §	29 (23-35)	30 (22-36)	29 (23-37)	0.909
Female ^†^	66.7	61.3	73.9	0.331
Time since diagnosis, years §	16 (10-22)	15 (10-22)	17 (9-24)	0.846
Basal glucose, mg/dL §	131 (93-202)	129 (88-235)	131 (98-196)	0.568
HbA1c, % §	9.0 (7.7-10.8)	8.9 (7.4-10.1)	9.8 (7.7-11.2)	0.168
Insulin doses, UI/kg/day §	0.74 ± 0.29	0.73 ± 0.31	0.75 ± 0.28	0.591
Overweight or obesity ^†^	55.0	56.2	59.1	0.771
Hypertension ^†^	18.4	19.4	17.4	0.999
Dyslipidemia ^†^	20.4	19.4	39.1	0.382
Chronic kidney disease ^†^	14.0	9.7	21.7	0.272
Neuropathy ^†^	20.4	12.9	30.4	0.177
Other autoimmune diseases ^†^	42.6	6.6	40.9	0.861
Tobacco use ^†^	5.6	0	13.6	0.076
FSSG >8 points†	28.0	0	82.6	NA
CQD >4 pOINTS ^†^	39.0	0	78.3	NA

* Positive for one or both questionnaires

** p value comparing patients with and without GERD symptoms using Mann-Whitney U test or Student's t test according data distribution.

§ Results are expressed as median (interquartile ranges) or means ± standard deviation.

†: value in %

NA = not applied.

Two-thirds of the patients were in intensive insulin treatment (basal / bolus) with an average of 40 ± 15 units per day, which represents 0.74 ± 0.29 IU/kg. Only 17% of the patients had a dose greater than 1 IU/kg of weight. Four percent were on treatment with insulin pump. Despite intensive care, 14% had HbA1c <7%, 43% had HbA1c <8% (moderate control) and the rest were above this level. In addition, 36% were on lipid control goals (defined as total cholesterol <200 mg/dL, LDL-c <100 mg/dL and triglycerides <150 mg/dL). Additionally, one fifth of the patients had antihypertensive treatment. Mean BMI was 26.7 ± 5.1 kg/m^2^, 45% had normal weight, 34% were overweight and 21% were obese.

Only 8% of the patients had a previous diagnosis of GERD, none of the patients had a documented *Helicobacter pylori* infection and 15% reported chronic use of antacid treatment.

### Questionnaire results 

Thirty-nine percent of patients presented GERD-compatible symptoms using the CDQ and 28% using the FSSG questionnaire. Fourteen percent had both positive questionnaires, 8% only one positive questionnaire and 32% of the patients had both questionnaires negative. The median score using the FSSG questionnaire was 5 points (2 - 13 points) and for the CDQ the median was 1 point (0-5 points). 

Considering the cutoff points reported in the literature (more than 8 points for FSSG and more than 4 points for CDQ), 39% had GERD symptoms using the FSSG and 29% using the CDQ. The kappa concordance test between the two questionnaires was 65% (*p* <0.001). Patients with GERD according to CDQ had higher HbA1c levels, compared to patients with negative questionnaires (10.7% vs. 8.6%, *p*= 0.022). In addition, smokers were more likely to be positive (*p*= 0.035).

We calculate sensitivity and specificity for different scores in both questionnaires, using a ROC curve. The FSSG questionnaire score of 11 points had a sensitivity of 71%, specificity of 32% and an area under the curve (AUC) of 0.514. A score of 8 points (as described in the literature) had a sensitivity of 71%, but the specificity decreased to 19%. A CDQ score of 7 points had a sensitivity of 83%, a specificity of 13% and an AUC of 0.441; meanwhile a score of 4 points (as described in the literature) had a sensitivity of 83%, with specificity of 3%.

On the other hand, patients with neuropathy were more frequently classified as positive for GERD using the FSSG (*p*= 0.030). Other clinical or biochemical parameters were not different among patients with or without GERD symptoms, as shown in Table 1.

The stratified analysis comparing patients with and without symptoms with one or both positive questionnaires showed that the groups were similar in age, duration of diabetes, BMI, insulin dose and glycemic control.

## Discussion 

Gastroesophageal reflux is common in the general population and its frequency varies among countries and ethnic groups depending on their diet and other biological and cultural factors. In Mexico, published studies show a prevalence of GERD from 11.9% to 35.0%, depending on the diagnostic method used ^18^.

Diabetes is one of the major risk factors for gastrointestinal diseases, including GERD. However, this disease can be associated with other pathologies such as neuropathy, infections and neoplasia. Since an extensive workup for GERD is costly, time-consuming and sometimes unsuccessful, validated screening tools are needed for each population to determine which patients are candidates for additional tests (eg, endoscopy). Clinical scales for GERD have been widely used and validated in different populations ^19^ ^-^ ^21^ including patients with diabetes. However, there is little information on the use of these tools in patients with T1D in Mexico.

Regarding this point, a recently published meta-analysis by Sun *et al*. ^22^, showed that diabetes increases the risk of GERD with an OR of 1.61 (1.36-1.91, *p*= 0.006). However, as specified by the authors, none of the manuscripts used for this analysis distinguish between patients with T1D or T2D. Patients with T1D are rarely studied as a separate population and this is especially important in adult patients that had diabetes for a long time and may have more severe comorbidities. Our investigation group considers that distinguishing these patients from other types of diabetes is important because, as we see in the previous results, most adult patients with T1D are very young (around 30 years of age) and they present with multiple complications and even incapacities, when they should be living normal lives with a better quality of life. Gastroesphageal diseases may be one of the contributing factors for poor metabolic control and quality of life, however these asseverations require further studies. 

Although the phenotype of hyperglycemia is similar in patients with T1D and T2D, patients living with T1D have a higher frequency of autoimmune diseases ^23^ and their manifestations may be confused with GERD. In this study we found that patients without GERD had lower frequencies of autoimmune diseases (6%) compared to patients with GERD (41%), however this difference did not reach statistical significance. 

 Another important issue is that nowadays there are several available questionnaires that evaluate the presence of gastrointestinal symptoms. The use of each questionnaire may have pros and cons. Selecting one questionnaire over the other requires careful examination of the characteristics of the evaluated group and the questionnaire's properties and previous validation in similar populations. In this study, we found that both questionnaires detected GERD with similar frequency, with a concordance of 65%. This concordance is greater than that reported among other questionnaires. Contreras-Omaña *et al*. ^24^, compared the CDQ questionnaire with the GQQ (GERD-DQ) questionnaire, which is also widely used to assess GERD. In this study, both questionnaires were applied in 220 individuals, of whom 57% were men, with a mean age of 38 years and 52% were overweight or obese. Initially, patients reported that the GQQ questionnaire was more difficult to understand and answer. In this population, 50% of the patients with GERD had at least one questionnaire positive, 45% were positive with CDQ, 23% were positive with GQQ and only 20% had both questionnaires positive. The authors propose that GQQ may be more useful in overweight patients and that the lack of correlation between the two questionnaires is due to the different parameters evaluated.

Despite those differences, no study has demonstrated that a questionnaire is superior to another to assess the symptoms of GERD, since they were designed for different purposes in different populations. However, the use of multiple scales increases the accuracy of the results. The CDQ was created by first-contact physicians and has the advantage of being easy to apply and has been validated in different populations; however it has a relatively complex scoring system and the limitation of not being able to be used if the patient is under treatment ^25^. In Mexico, Gómez-Escudero et al. ^14^, assessed the usefulness of the CDQ questionnaire in patients who complained of heartburn twice a week during the three months prior to the study. They considered the GERD questionnaire as positive with a score >4 (the diagnosis was corroborated with endoscopy data and 24 h pHmetry). The study included 125 patients, 65% were women with a mean age of 48 years, and found that 86% of the patients had a score >4. When the CDQ was compared with 24 h pHmetry it reached a sensitivity of 89%, specificity of 23%, positive predictive value of 55% and negative predictive value of 61%. When they compared with endoscopic evidence of esophagitis, sensitivity increased to 94% and the predictive value increases to 90%. The limitation of this study is that the studied patients were selected for their symptoms which may increase the sensitivity of the test. 

The FSSG questionnaire has been used in different ethnic groups, correlating adequately with endoscopic findings and has the advantage of being able to be used in patients undergoing treatment ^25^ ^,^ ^26^. Miyamoto *et al*.^27^, studied 255 patients with GERD who completed the FSSG questionnaire and performed an endoscopy. After treatment with a proton pump inhibitor (PPI, rabeprazole 10 mg/day) for 3 to 6 months, patients were invited to choose between four options: continue treatment; continue with the inhibitor and add a prokinetic; change to a histamine (H2) receptor antagonist or to discontinue treatment. After treatment, the total FSSG score decreased at both 3 and 6 months. However, when dividing by reflux-related (RS) or dyspepsia (DS) scores, they found that RS ≥7 had an OR of 2.0 (95% CI: 1.2-3.4) to continue treatment with PPI, whereas RS ≤6 had an OR of 1.7 (95% CI: 1.1-2.9) for cessation of the drug. These authors conclude that FSSG can predict which patients may require maintenance therapy ^27^.

In this study, we found that a large proportion of patients with T1D had GERD symptoms detected by both questionnaires. Despite this many had not been previously evaluated by a gastroenterologist and had received empiric and irregular treatment with proton pump inhibitors or H2 blockers. It is interesting to note that patients classified as positive (with one or both questionnaires) were not different from patients with negative results with respect to time of diagnosis or glycemic control. In addition, other clinical, physical or biochemical characteristics were not different. Those findings could imply that although diabetes and its comorbidities increase the chances of having symptoms of GERD, they are not the only factors that influence its presence or severity. It could also mean that clinical assessment is not sufficient to determine which patients are at increased risk for GERD and that special questionnaire and routine evaluations by specialists are needed, particularly in those patients who are symptomatic. Finally, we found that the cutoff points proposed by the original authors lack specificity in these patients, and in order to achieve similar sensitivities and specificities, patients with T1D may need to have higher scores to suspect GERD, this may be due to other comorbidities or the fact that many patients were being empirically treated with antacid therapies. 

Faria *et al*. ^28^, evaluated the GERD symptoms in Brazilian patients with T1D using the ROMA III criteria and confirmed them with endoscopic evaluation and pathology. They found a high frequency of *H. pylori* infection as well as increased prevalence of GERD symptoms in patients with T1D in comparison with healthy controls. We should remember that our population has previously reported high prevalences of *H. pylori* infection. They also found a correlation with HbA1c and other parameters of glycemic control and anthropometry. Similar to our study, they found a lack of association between the time since diagnosis, the presence of diabetic comorbidities and GERD symptoms. Finally, they concluded that patients with T1D had an increase in the prevalence of GERD due to a possible relationship with other related pathologies.

The limitations of our study include the small number of patients whose diagnosis was corroborated by endoscopy and the absence of esophageal pHmetry determinations. However, our goal was to evaluate the utility of two clinical scales to detect symptoms of GERD in order to avoid invasive procedures such as endoscopy. We believe that in developing countries, where access to specialized medical care is limited as well as funding for research, the use of cheap and simple tools such as these questionnaires is especially important in order to detect those patients that require additional care. The combination of at least two questionnaires may increase the positive predictive value, but this needs further evaluation. We probed that CDQ and FSSG are useful for detecting GERD symptoms in patients with T1D and considering the high frequency of symptomatic patients, we suggest that additional research is needed in this group of patients to determine the most effective and efficient techniques for diagnosis in order to prevent future gastrointestinal complications and reduce related costs.

## Conclusion

CDQ and FSSG questionnaires are useful for identifying GERD symptoms in patients with T1D. In this population, the FSSG questionnaire had greater power of detection for GERD in comparison with CDQ. We suggest that patients with T1D who are positive for one or both questionnaires require corroboration by endoscopic study and assessment by a gastroenterologist.

## Annexes

**Annex 1:** FSSG questionnaire and score. ^(10)^ Questions 2, 3, 5, 8 and 11 refer to dyspepsia symptoms, rest are questions about reflux symptoms.

Questions	Frequency				
	Never	Occasionally	Sometimes	Often	Always
1. Do you get heartburn?	0	1	2	3	4
2. Does your stomach get bloated?	0	1	2	3	4
3. Does your stomach ever feel heavy after meals?	0	1	2	3	4
4. Do you sometimes subconsciously rub your chest with your hand?	0	1	2	3	4
5. Do you ever feel sick after meals?	0	1	2	3	4
6. Do you get heartburn after meals?	0	1	2	3	4
7. Do you have an unusual (e.g. burning) sensation in your throat?	0	1	2	3	4
8. Do you feel full while eating meals?	0	1	2	3	4
9. Do some things get stuck when you swallow?	0	1	2	3	4
10. Do you get bitter liquid (acid) coming up into your throat?	0	1	2	3	4
11. Do you burp a lot?	0	1	2	3	4
12. Do you get heartburn if you bend over?	0	1	2	3	4
Total					

## Annex

**Annex 2:** Carlsson-Dent Questionnaire ^(14)^

Question	Score			
1. Which of the following sentences best describes your main complaint? Mark an option.	Burning sensation or burning pain that starts in the pit of your stomach or chest and goes up into your throat (heartburn)			+5
	Nausea or vomiting			0
	Pain in the middle of your chest when you eat			+2
	None of the above			0
2. Which of the following sentences best describes the time at which you have the complaint? Mark an option	At any time and there is no relation to eating (neither improves or worsens with meals)			-2
	Within the first 2 hours after eating			+3
	Always occurs at the same time of day or night and is not related to eating			0
3. What happens to your complaint in the following situations: does it get worse, get better, or nothing happens? Read each sentence and circle what happens to your main complaint		It gets worse	It gets better	No effect
	You eat a lot or more than you are accustomed to	+1	-1	0
	You eat fatty foods	+1	-1	0
	You eat spicy or very seasoned foods	+1	-1	0
4. What happens to your main complaint when you take antacids? Mark an option.	Nothing			0
	Complete relief within the first 15 minutes of having taking them			+3
	Complete relief 15 minutes after taking them			0
	I don't take antacids			0
5. What happens to your main complaint when you bend over or lie down? Mark an option	Nothing			0
	It gets worse or the activity causes it			+1
	It gets better			-1
	I don't know			0
6. Which of the following options best describes the effect that carrying heavy things, straining, or doing anything strenuous has on your main complaint?	No effect			0
	It gets worst or the activity causes it			+1
	It gets better			-1
	I don't know or I don't do strenuous things			0
7. If you regurgitate (the food in your stomach returns to your throat), what happens to your main complaint?	Nothing			0
	It gets worse or the regurgitation causes it			+2
	It gets better			-1
	I don't know or I don't regurgitate			0
