# Supplementary material for: Gastroesophageal reflux disease in patients with long standing type 1 diabetes mellitus: utility of two self-report questionnaires in a multifactorial disease
Source: Colomb Med (Cali). 2017 Sep 30;48(3):132–7. doi: 10.25100/cm.v48i3.2801 (PMC5687865; doi:10.25100/cm.v48i3.2801)
Supplement: Translation

# Translation: Enfermedad por Reflujo Gastroesofágico en pacientes con Diabetes Mellitus tipo 1: utilidad de dos cuestionarios auto-aplicables

## Introducción

La Enfermedad por Reflujo Gastroesofágico (ERGE) se produce cuando el reflujo del contenido del estómago ocasiona síntomas que deterioran la calidad de vida de los pacientes o provocan complicaciones. Tiene una prevalencia del 10 al 40% en la población adulta, pero varía de acuerdo a las características de la población, la dieta y la presencia de comorbilidades ^1^. Las enfermedades gastrointestinales son comunes en los pacientes con diabetes mellitus, así como los síntomas de disfagia, acidez y regurgitación ^2^. La importancia de detectar la ERGE en estos pacientes radica en que la enfermedad disminuye la tolerancia a los hipoglucemiantes orales y disminuye la calidad de vida ^3^. Además, debido a lo inespecífico de los síntomas, puede pasar desapercibida por muchos años.

La identificación adecuada de los síntomas de ERGE ^4^ en los pacientes con diabetes tipo 1 (DM1) es complicada, debido a la larga lista de patologías que pueden desarrollar estos pacientes y que pueden confundir el diagnóstico; además, muchas de las comorbilidades microvasculares y las complicaciones crónicas tienen un impacto en la sintomatología gástrica ^5^. De hecho, la neuropatía autonómica es uno de los problemas principales, ya que se presenta en los pacientes con larga evolución de la enfermedad a pesar del control glucémico, es altamente prevalente e irreversible. 

Por otra parte, los pacientes con DM1 pueden desarrollar otras enfermedades como la gastritis autoinmune, la enfermedad celíaca o la enfermedad de Crohn cuyos síntomas se confunden con los de ERGE. El estudio diferencial de estas patologías incluye endoscopias, identificación sérica de autoanticuerpos y el cumplimiento de algoritmos específicos de diagnóstico realizados por gastroenterólogos especializados. Estos estudios pueden ser molestos para el paciente, costosos y en algunos países existen limitaciones para su solicitud. Por ello sería más práctico descartar en primer lugar el diagnóstico de ERGE mediante una herramienta clínica eficaz y de fácil aplicación. 

Ya que el estudio de ERGE no está disponible en todas las regiones de nuestro país, los gastroenterólogos sugieren que el uso de datos clínicos pueden ser buenos indicadores de ERGE. Con el paso del tiempo, se han elaborado escalas clínicas para ayudar a la adecuada identificación de la enfermedad. Carlsson et al., desarrollaron un cuestionario (CDQ) y validaron sus hallazgos con endoscopia y pHmetría de 24 horas ^6^. Este cuestionario es auto-diligenciado y un puntaje mayor a cuatro tiene una sensibilidad del 92% y una especificidad del 19% para el tamizaje de ERGE. Después, Kusano et al., desarrollaron otro cuestionario llamado FSSG (Frequency Scale for the Symptoms of GERD), argumentando que el CDQ sólo implica que un evento es más típico de ERGE que otras condiciones, pero no exclusivo y no es útil para evaluar la gravedad del ERGE o la respuesta a tratamiento. Un puntaje de 8 en la escala FSSG, tiene sensibilidad del 62% y especificidad del 59% para el diagnóstico de ERGE, corroborado mediante endoscopia ^7^. 

Ambos cuestionarios han sido ampliamente usados y validados en diferentes poblaciones, incluyendo pacientes de nuestro país ^8^^-^^10^ e inclusive en pacientes con Diabetes Mellitus tipo 2 ^11^^,^^12^; sin embargo, no han sido utilizados en los pacientes con DM1. El objetivo de este estudio fue evaluar la prevalencia y gravedad de la ERGE en paciente con DM1 utilizando una herramienta sencilla y auto-diligenciada. 

## Materiales y Métodos

Se realizó un estudio transversal, analítico, en un grupo de pacientes seleccionados al azar de la Clínica de DM1 del Hospital de Especialidades de Centro Médico Nacional Siglo XXI del Instituto Mexicano del Seguro Social (IMSS) de febrero a mayo del 2015. Todos los pacientes cumplieron los criterios de la American Diabetes Association (ADA) para el diagnóstico de DM1 ^13^: se detectaron autoanticuerpos anti-GAD65 o anti-IA2 y todos tenían péptido C por debajo de los límites inferiores para nuestra población ^13^; en este estudio no se restringió su selección por edad o por peso. 

Para su inclusión debían de tener un tiempo de diagnóstico de al menos cinco años, capacidad visual para leer las preguntas del cuestionario y al menos escolaridad primaria para comprender el cuestionario sin ayuda. Se excluyeron a los pacientes con antecedente de cirugía gástrica, diagnóstico previo por endoscopia, pérdida de peso no intencionada, disfagia severa o progresiva o hemorragia gastrointestinal. Se registraron los datos clínicos y bioquímicos al momento de la valoración inicial. Todos los pacientes completaron los cuestionarios FSSG ^10^ (Anexo 1) y CDQ ^14^ (Anexo 2) validados en español. Un puntaje mayor a 4 en el cuestionario de CDQ o un puntaje mayor a 8 en el FSSG fueron considerados como positivos para la detección de ERGE. Todos los cuestionarios fueron aplicados por el investigador principal (EVS) y analizados por un investigador cegado (CRR) con una κ= 0.7 (concordancia), *p:* < 0.001.

El protocolo fue aprobado por el Comité Local de Investigación y Ética en Investigación en Salud. Los objetivos del estudio fueron ampliamente explicados a los participantes, quienes dieron su consentimiento informado por escrito. Los procedimientos respetaron las normas éticas de acuerdo a la Declaración de Helsinki de 1975, revisada en 2013. 

### Evaluación bioquímica

Se solicitaron estudios de laboratorio a los participantes después de un periodo de 8 horas de ayuno. Se determinó glucosa, colesterol, colesterol HDL (c-HDL) y triglicéridos, mediante técnica de espectrofotometría ^15^, utilizando kits comerciales. Para obtener la concentración de c-HDL, se utilizó la prueba enzimática de precipitación con polietilenglicol y dextrán sulfato ^16^ y fueron analizados con la misma técnica fotocolorimétrica del colesterol. La hemoglobina glucosilada (HbA1c) fue analizada mediante inmunoanálisis. La concentración de colesterol LDL (c-LDL) fue calculada mediante la fórmula de Friedewald ^17^.

### Análisis estadístico

Los datos fueron analizados utilizando el paquete estadístico STATA (r) versión 11. Se utilizó la prueba de Kolmogorov-Smirnov para valorar normalidad. Los resultados son expresados como medias y desviaciones estándar (DE) o medianas con rangos intercuartílicos (RI), de acuerdo a la distribución de los datos. Los resultados fueron evaluados mediante un análisis estratificado, buscando asociaciones entre las variables cuantitativas mediante la prueba de U de Mann-Whitney o t de Student y para variables cualitativas mediante χ^2^ o prueba de Fisher. Se realizaron curvas ROC (Receiver Operating Characteristics) de las diferentes escalas para obtener el mejor punto de corte (con intervalos de confianza del 95%) para la detección de ERGE.^.^ Se consideró *p* < 0.05 como estadísticamente significativo. 

## Resultados

### Características basales

Cincuenta y cuatro pacientes cumplieron con los criterios de inclusión y completaron el cuestionario, 36 mujeres (67%). La mediana de edad del grupo estudiado fue de 29 años (23-35 años), mientras que el tiempo medio de evolución fue de 16 años (rangos 10-22 años). Todos los pacientes tuvieron más de 5 años de diagnóstico de diabetes tipo 1 y 72% más de 10 años de diagnóstico (Tabla 1). Las comorbilidades asociadas fueron: otra enfermedad autoinmune (43%), principalmente hipotiroidismo; 20.4% tuvieron dislipidemia (previamente detectados con hipertrigliceridemia o niveles disminuidos de c-HDL de acuerdo a sexo o aquellos con un tratamiento establecido); 18.4% tuvieron diagnóstico previo de hipertensión y 14% presentaron enfermedad renal crónica (validada mediante la escala de KDOQI utilizando la tasa de filtración glomerular). 

**Tabla 1:** Características basales de los 54 pacientes estudiados con DM1, con y sin síntomas de ERGE.

Variable	Población total (n= 54)	Sin síntomas de ERGE (n= 32)	Positivo* (n= 22)	p**
Edad, años §	29 (23-35)	30 (22-36)	29 (23-37)	0.909
Mujeres ^†^	66.7	61.3	73.9	0.331
Tiempo de diagnóstico, años §	16 (10-22)	15 (10-22)	17 (9-24)	0.846
Glucosa basal, mg/dL §	131 (93-202)	129 (88-235)	131 (98-196)	0.568
HbA1c §	9.0 (7.7-10.8)	8.9 (7.4-10.1)	9.8 (7.7-11.2)	0.168
Dosis de insulina, UI/kg/día §	0.74 ± 0.29	0.73 ± 0.31	0.75 ± 0.28	0.591
Sobrepeso u obesidad ^†^	55.0	56.2	59.1	0.771
Hipertensión ^†^	18.4	19.4	17.4	0.999
Dislipidemia ^†^	20.4	19.4	39.1	0.382
Enfermedad Renal Crónica ^†^	14.0	9.7	21.7	0.272
Neuropatía ^†^	20.4	12.9	30.4	0.177
Otras enfermedades autoinmunes ^†^	42.6	6.6	40.9	0.861
Uso de tabaco ^†^	5.6	0	13.6	0.076
FSSG >8 puntos ^†^	28.0	0	82.6	NA
CQD >4 puntos ^†^	39.0	0	78.3	NA

* Positivo para 1 o ambos cuestionarios

**Valor de p al comparar aquellos pacientes positivos para síntomas de ERGE con aquellos pacientes sin síntomas usando U de Mann-Whitney o t de student de acuerdo a los datos.

§: Los resultados se expresan como medianas (Rangos intercuartílicos) o medias ± Desviación Estándar.

†: Valores en %

NA = No aplica.

Dos tercios de los pacientes utilizaron un régimen intensivo (basal/bolos) con un promedio de 40 ± 15 unidades por día, que representa una media de 0.74 ± 0.29 UI/kg. Solo 17% de los pacientes tuvieron una dosis mayor a 1 UI/kg de peso. El 4% estaban en tratamiento con bomba de insulina. A pesar de la terapia intensiva, 14% presentaron HbA1c <7%, 43% estaban con HbA1c <8% (control moderado) y el resto estaban por arriba de este nivel. Además, 36% estaban en metas para el control de lípidos en pacientes diabéticos (colesterol total <200 mg/dL, c-LDL a <100 mg/dL y triglicéridos <150 mg/dL) . Adicionalmente, un quinto de los pacientes tuvieron tratamiento antihipertensivo. 

El IMC reportado fue de 26.7 ± 5.1 kg/m^2^, 45% de los pacientes presentaron peso normal, 34% sobrepeso y 21% obesidad.

Solo el 8% de los pacientes tuvieron diagnóstico previo de ERGE, ningún paciente presentó infección por *Helicobacter pylori* y 15% reportaron uso crónico de tratamiento antiácido. 

### Resultados del cuestionario

Los pacientes que presentaron síntomas compatibles con ERGE utilizando el CDQ fue de 39% y 28% utilizando el cuestionario FSSG;.para ambos cuestionarios positivos se presentaron el 14% de los pacientes, 8% sólo un cuestionario positivo y 32% de los pacientes tenían ambos cuestionarios negativos. La mediana de puntaje utilizando el cuestionario FSSG fue de 5 puntos (2-13 puntos) y para el CDQ la mediana fue de 1 punto (0-5 puntos).

Considerando los puntos de corte reportados en la literatura (más de 8 puntos para FSSG y más de 4 puntos para CDQ), 39% tenían síntomas de ERGE utilizando el FSSG y 29% utilizando el CDQ. El test de concordancia kappa entre ambos cuestionarios fue del 65% (*p* <0.001). Los pacientes con ERGE de acuerdo al CDQ tenían medianas de concentraciones de HbA1c más altas, en comparación con los pacientes con cuestionarios negativos (10.7% vs. 8.6%, *p*= 0.022). Además, los fumadores eran más propensos a resultar positivos (*p*= 0.035).

Al realizar curvas ROC se encontraron sensibilidades y especificidades para diferentes puntajes en el cuestionario. Con el cuestionario FSSG en 11 puntos se encontró una sensibilidad del 71%, una especificidad del 32% y un área bajo la curva (AUC) de 0.514. Mientras que un puntaje de 8, la sensibilidad fue del 71%, pero la especificidad disminuyó al 19%. Con el CDQ, a 7 puntos se tiene una sensibilidad del 83%, una especificidad del 13% y una AUC de 0.441; mientras que con 4 puntos (descritos en la literatura) se alcanzó una sensibilidad del 83%, con especificidad del 3%.

Por otra parte, los pacientes con neuropatía fueron más frecuentemente clasificados como positivos para ERGE utilizando el FSSG (*p*= 0.030). También se observó que los pacientes fumadores tenían mayor frecuencia de sintomatología (*p*= 0.04). Otros parámetros clínicos o bioquímicos no fueron diferentes entre los pacientes con o sin síntomas de ERGE, como se observa en la Tabla 1.

El análisis estratificado comparando los pacientes con y sin síntomas con uno o ambos cuestionarios positivos, mostró que los grupos eran similares en cuanto a edad, tiempo de evolución de diabetes, IMC, dosis de insulina y control glucémico. 

## Discusión

El reflujo gastroesofágico es común en la población general y su frecuencia varía entre los países y grupos étnicos dependiendo de su dieta y otros factores biológicos y culturales. En México, los estudios publicados muestran una prevalencia de ERGE de entre 11.9% a 35%, dependiendo del método de diagnóstico empleado ^18^.

La diabetes es uno de los mayores factores de riesgo para enfermedades gastrointestinales, incluyendo la ERGE. Sin embargo, esta enfermedad se puede asociar con otras patologías como neuropatía, infecciones y neoplasias. Debido a que el diagnóstico completo de ERGE es costoso, consume tiempo y en ocasiones es infructuoso, se necesitan herramientas de detección validadas para cada población, que dictaminen qué pacientes son candidatos para un abordaje adicional (por ejemplo con estudios de endoscopia). Las escalas clínicas para ERGE han sido ampliamente usadas y validadas en diferentes poblaciones ^19^^-^^21^, incluyendo pacientes diabéticos; sin embargo, existe poca información del uso de estas herramientas en el paciente con DM1 en México. 

Con respecto a esto, un meta-análisis recientemente publicado por Sun *et al*. ^22^, mostraron que la diabetes es un factor importante que incrementa el riesgo de ERGE con un OR de 1.61 (IC 95%: 1.36-1.91, *p*= 0.006); sin embargo, tal como lo especifican los autores, ninguna de las publicaciones utilizadas para este análisis hace la distinción entre pacientes con DM1 o DM2. Los pacientes con DM1 raramente son estudiados como una población por separado, especialmente tratándose de pacientes adultos, con largo tiempo de evolución y con comorbilidades más severas. Si bien el fenotipo de hiperglucemia es similar en los pacientes con DM1 y DM2, los pacientes que viven con DM1 tienen mayor frecuencia de enfermedades autoinmunes ^23^ cuyas manifestaciones pueden ser confundidas con ERGE. En este estudio, encontramos que ambos cuestionarios detectaron ERGE con frecuencia similar, con una concordancia del 65%. Esta concordancia es mayor que la reportada entre otros cuestionarios. Contreras-Omaña *et al*. ^24^, compararon el cuestionario CDQ con el cuestionario GQQ (GERD- DQ), el cual también es ampliamente utilizado para valorar ERGE. En este estudio se aplicaron ambos cuestionarios en 220 individuos, de los cuales 57% eran hombres, con media de edad de 38 años y 52% tenían sobrepeso u obesidad. De forma inicial, los pacientes reportaron que el cuestionario GQQ es más difícil de comprender y responder. En esta población 50% de los pacientes con síntomas de ERGE tuvieron al menos un cuestionario positivo, 45% fueron positivos con el CDQ, 23% positivos con el GQQ y solo 20% tuvieron positivos ambos cuestionarios. Los autores proponen que el GQQ puede tener mayor utilidad en pacientes con sobrepeso y que la falta de correlación entre ambos cuestionarios se debe a que evalúan parámetros distintos.

A pesar de estas diferencias, no existen estudios que demuestren que un cuestionario es superior a otro para valorar los síntomas de ERGE, ya que fueron diseñados para propósitos diferentes en poblaciones diferentes; sin embargo, el uso de múltiples escalas incrementa la exactitud de los resultados. El CDQ fue creado para y por médicos de primer contacto, tiene la ventaja de ser fácil de aplicar y ha sido validado en diferentes poblaciones; sin embargo, tiene un sistema de puntaje relativamente complejo y la limitante de no poder ser utilizado si el paciente está en tratamiento ^25^. En México, Gómez-Escudero *et al*. ^14^, evaluaron la utilidad del cuestionario CDQ en pacientes con pirosis, dos veces por semana durante los tres meses previos a la realización del estudio, con una panendoscopia en las ocho semanas previas y consideraron positivo el cuestionario para ERGE con un puntaje >4 (el diagnóstico se corroboró con los datos endoscópicos y con pHmetría de 24 horas). El estudio incluyó 125 pacientes, 65% de los cuales fueron mujeres, con edad promedio de 48 años y encontraron que el 86% de los pacientes tuvieron un puntaje >4. Al compararse el CDQ con la pHmetría alcanzó una sensibilidad del 89%, especificidad del 23%, valor predictivo positivo de 55% y valor predictivo negativo de 61%; mientras que al compararse con la evidencia endoscópica de esofagitis, la sensibilidad aumentó al 94% y el valor predictivo positivo al 90%. 

El cuestionario FSSG ha sido utilizado en diferentes grupos étnicos con buenos resultados, con buena correlación entre el puntaje y los hallazgos endoscópicos y tiene la ventaja de poder ser utilizado en los pacientes en tratamiento ^25^^,^^26^. Miyamoto *et al*.^27^, estudiaron a 255 pacientes con ERGE en quienes aplicaron el cuestionario FSSG e hicieron endoscopia. Después de tratamiento con un inhibidor de la bomba de protones (IBP. rabeprazol 10 mg/día) durante 3 a 6 meses, los pacientes fueron invitados a escoger entre cuatro opciones: continuar el tratamiento, continuar con el inhibidor y agregar un procinético, cambiar a un antagonista del receptor de histamina H2 o bien, descontinuar el tratamiento. Después del tratamiento, el puntaje total del FSSG disminuyó tanto a los 3 como a los 6 meses; sin embargo, al dividirlo por puntaje relacionado con reflujo (RS) o con dispepsia (DS), encontraron que el RS ≥7 tiene un OR de 2.0 (IC 95%: 1.2-3.4) para continuar el tratamiento con IBP, mientras que el RS ≤6 tuvo un OR de 1.7 (IC 95%: 1.1-2.9) para el cese del medicamento. Estos autores concluyen que el FSSG puede predecir qué pacientes pueden requerir terapia de mantenimiento ^27^. 

En este estudio, encontramos que una gran proporción de pacientes con DM1 tenían síntomas de ERGE detectados mediante ambos cuestionarios; a pesar de esto, muchos no habían sido previamente evaluados por un gastroenterólogo y habían recibido tratamiento empírico e irregular con inhibidores de la bomba de protones o bloqueadores H2. Es interesante mencionar que los pacientes clasificados como positivos (con uno o los dos cuestionarios) no fueron diferentes de los pacientes con resultados negativos, con respecto al tiempo de diagnóstico o el control glucémico. Además, otras características clínicas, físicas o bioquímicas no fueron diferentes; lo cual podría traducirse en que si bien la diabetes y sus comorbilidades incrementan las posibilidades de tener síntomas de ERGE, no son el único factor que influye en su presencia o severidad. También puede significar que la valoración clínica no es suficiente para determinar que pacientes tienen mayor riesgo de presentar ERGE y que se necesitan cuestionarios especiales y evaluaciones de rutina por especialistas, sobre todo en aquellos pacientes sintomáticos. 

Faria *et al*. ^28^, evaluaron los síntomas de ERGE en pacientes brasileños con DM1 utilizando los criterios de ROMA III, confirmado con evaluación endoscópica y patología. Ellos encontraron una elevada frecuencia de infección por *H. Pylori*, así como incremento en la prevalencia de síntomas de ERGE en pacientes con DM1 en comparación con controles sanos y una posible correlación con HbA1c y otros parámetros de control glucémico y antropometría. En concordancia con nuestro estudio, también encontraron falta de asociación entre el tiempo de diagnóstico, la presencia de comorbilidades diabéticas y los síntomas de ERGE. Finalmente, concluyeron que los pacientes con DM1 tenían incremento en la prevalencia de ERGE por una posible relación con otras patologías asociadas a DM1. 

Entre las limitaciones de este estudio, radica el escaso número de pacientes en quienes se corroboró el diagnóstico mediante endoscopia y la ausencia de determinaciones de pHmetría esofágica; sin embargo, nuestro objetivo era evaluar la utilidad de dos escalas clínicas para detectar síntomas de ERGE con el fin de evitar procedimientos invasivos como la endoscopia. Nosotros consideramos que en países en vías de desarrollo, donde el acceso a atención médica especializada es limitado y la obtención de fondos para investigación es difícil, el uso de herramientas económicas y sencillas como estos cuestionarios, son especialmente útiles para detectar qué pacientes requieren atención adicional o inmediata. La combinación de al menos dos cuestionarios puede incrementar el valor predictivo positivo, lo cual aún necesita ser evaluado. Consideramos que el CDQ y el FSSG son útiles para detectar síntomas de ERGE en pacientes con DM1 y, considerando la elevada frecuencia de pacientes sintomáticos, sugerimos que se necesita investigación adicional en este grupo de pacientes para determinar cuáles son las técnicas más efectivas y eficaces para el diagnóstico, con la finalidad de prevenir futuras complicaciones gastrointestinales y disminuir los costos relacionados.

## Conclusiones

Los cuestionarios CDQ y FSSG son útiles para identificar síntomas de ERGE en pacientes con DM1. En esta población, el cuestionario FSSG tuvo mayor poder de detección de ERGE en comparación con el CDQ. Sugerimos que los pacientes con DM1 que resulten positivos para uno o ambos cuestionarios, requieren corroboración mediante estudio endoscópico y valoración por un gastroenterólogo. 

## Anexos

**Anexo 1:** Cuestionario y puntaje de la escala FSSG ^(10)^. Las preguntas 2, 3, 5, 8 y 11 se refieren a síntomas de dispepsia, el resto son preguntas sobre síntomas de reflujo.

Preguntas	Frecuencia				
	Nunca	Raramente	A veces	Frecuentemente	Siempre
1. ¿Tiene sensación de ácidez en la boca del estómago?	0	1	2	3	4
2. ¿Su estómago se inflama?	0	1	2	3	4
3. ¿Siente el estómago pesado después de comer?	0	1	2	3	4
4. ¿Ha notado si en ocasiones, de manera subconsciente frota su pecho con la mano?	0	1	2	3	4
5. ¿Se siente enfermo después de comer?	0	1	2	3	4
6. ¿Tiene acidez después de los alimentos?	0	1	2	3	4
7. ¿Tiene sensación de quemadura en la garganta?	0	1	2	3	4
8. ¿Se siente lleno mientras está comiendo?	0	1	2	3	4
9. ¿Algunos alimentos se le quedan adheridos/pegados al tragarlos?	0	1	2	3	4
10. ¿Siente líquido ácido/amargo que sube por su garganta?	0	1	2	3	4
11. ¿Eructa frecuentemente?	0	1	2	3	4
12. ¿Tiene acidez si se agacha?	0	1	2	3	4
Total					

## Anexos

**Anexo 2:** Cuestionario de Carlsson-Dent^(14)^

Pregunta	Puntaje			
1. ¿Cuál de los siguientes enunciados describe mejor su molestia principal? (marque solo una opción)	Sensación de quemadura o ardor que inicia en la boca del estómago o en el pecho y sube hasta la garganta (agruras)			+5
	Náusea o vómito			0
	Dolor a la mitad del pecho cuando ingiere alimentos			+2
	No presenta ninguna de estas molestias			0
2. ¿Cuál de los siguientes enunciados describe mejor el momento en el que ocurre su molestia? (marque solo una opción)	En cualquier momento y no tiene relación con los alimentos (ni mejora ni empeora con las comidas)			-2
	En las primeras dos horas después de comer			+3
	Siempre ocurre a la misma hora del día o la noche, y no tiene relación con las comidas			0
3. ¿Qué pasa con sus molestias en las siguientes situaciones: empeora, mejora o no pasa nada? (lea cada enunciado y encierre en un círculo lo que sucede con su molestia principal)		Empeoran	Alivian	No tienen efecto
	Come abundantemente o más de lo acostumbrado	+1	-1	0
	Come alimentos grasos	+1	-1	0
	Come alimentos muy picantes o condimentados	+1	-1	0
4. ¿Qué pasa con su molestia principal cuando toma antiácidos? (marque solo una opción).	Nada			0
	Alivio definitivo en los primeros 15 minutos de haberlos tomado			+3
	Alivio definitivo después de 15 minutos de haberlo tomado			0
	No toma antiácidos			0
5. ¿Qué sucede con su molestia principal cuando se agacha o se acuesta? (marque solo una opción)	Nada			0
	La empeora o provoca su aparición			+1
	La mejora			-1
	No sabe definirlo con claridad			0
6. ¿Cuál de las siguientes opciones describe mejor el efecto de cargar cosas pesadas, pujar o realizar cualquier esfuerzo, sobre su molestia principal?	No tiene ningún efecto			0
	La empeora o provoca su aparición			+1
	La mejora			-1
	No sabe definirlo / No realiza estos esfuerzos			0
7. Si usted tiene regurgitaciones (siente que se regresan los alimentos del estómago a su garganta) ¿Qué pasa con su molestia principal?	Nada			0
	La empeora o provoca su aparición			+2
	La mejora			-1
	No sabe definirlo / No me sucede			0
